# Identification and Characterization of a Novel Epitope of ASFV-Encoded dUTPase by Monoclonal Antibodies

**DOI:** 10.3390/v13112175

**Published:** 2021-10-28

**Authors:** Shuai Zhang, Rui Wang, Xiaojing Zhu, Jiaxin Jin, Wenlong Lu, Xuyang Zhao, Bo Wan, Yifei Liao, Qin Zhao, Christopher L. Netherton, Guoqing Zhuang, Aijun Sun, Gaiping Zhang

**Affiliations:** 1 College of Veterinary Medicine, Henan Agricultural University, Zhengzhou 450046, China; onwardzs@163.com (S.Z.); ruiw_0623@163.com (R.W.); zhuxiaojing2020@163.com (X.Z.); jiaxinjin2020@163.com (J.J.); lwlhnnydx823@163.com (W.L.); zhaoxuyang17@163.com (X.Z.); wanboyi2000@163.com (B.W.); 2 International Joint Research Center of National Animal Immunology, College of Veterinary Medicine, Henan Agricultural University, Zhengzhou 450046, China; 3 Division of Infectious Disease, Department of Medicine, Brigham and Women’s Hospital, Harvard Medical School, Boston, MA 02115, USA; yliao8@bwh.harvard.edu; 4 Department of Preventive Veterinary Medicine, College of Veterinary Medicine, Northwest A&F University, Xianyang 712100, China; qinzhao_2004@nwsuaf.edu.cn; 5 The Pirbright Institute, Ash Road, Pirbright, Surrey GU24 0NF, UK; christopher.netherton@pirbright.ac.uk; 6 Key Laboratory of Animal Immunology, Ministry of Agriculture and Rural Afairs & Henan Provincial Key Laboratory of Animal Immunology, Henan Academy of Agricultural Sciences, Zhengzhou 450002, China

**Keywords:** African swine fever virus, African swine fever, dUTPase, epitope, therapeutic drug

## Abstract

Deoxyuridine 5′-triphosphate nucleotidohydrolase (dUTPase) of African swine fever virus (ASFV) is an essential enzyme required for efficient virus replication. Previous crystallography data have indicated that dUTPase (E165R) may serve as a therapeutic target for inhibiting ASFV replication; however, the specificity of the targeting site(s) in ASFV dUTPase remains unclear. In this study, 19 mouse monoclonal antibodies (mAbs) were produced, in which four mAbs showed inhibitory reactivity against E165R recombinant protein. Epitope mapping studies indicated that E165R has three major antigenic regions: 100–120 aa, 120–140 aa, and 140–165 aa. Three mAbs inhibited the dUTPase activity of E165R by binding to the highly conserved 149–RGEGRFGSTG–158 amino acid sequence. Interestingly, 8F6 mAb specifically recognized ASFV dUTPase but not Sus scrofa dUTPase, which may be due to structural differences in the amino acids of F151, R153, and F154 in the motif V region. In summary, we developed anti-E165R-specific mAbs, and identified an important antibody-binding antigenic epitope in the motif V of ASFV dUTPase. Our study provides a comprehensive analysis of mAbs that target the antigenic epitope of ASFV dUTPase, which may contribute to the development of novel antibody-based ASFV therapeutics.

## 1. Introduction

African swine fever virus (ASFV) is the causative agent of African swine fever (ASF), a highly contagious and hemorrhagic lethal porcine disease with up to 100% mortality [1]. First reported in Kenya in 1921, ASF has since spread to Europe and Latin America. In 2018, the first ASF outbreak in China became endemic in a short period, causing substantial economic losses [2]. Therefore, there is an urgent need to develop effective vaccines or drugs that can aid the prevention and control of ASF [3].

ASFV is the only member of the family *Asfarviridae*, genus *Asfivirus*. A mature ASFV particle contains an outer enveloped membrane, an icosahedral capsid, an inner membrane, an inner core-shell, and a double-stranded DNA genome [4,5,6]. ASFV encodes over 150 proteins, most of which are uncharacterized [7]. The product of the ASFV *E165R* gene is similar to that of *M. tuberculosis* deoxyuridine 5′-triphosphate nucleotidohydrolase (dUTPase) in terms of the overall protein structure and the presence of an active enzymatic center. Proteins with these characteristics are found to be commonly expressed in various living organisms and viruses. Located in the cytoplasm of infected cells [8], E165R maintains the fidelity of the viral genome during replication by orchestrating the ratio of deoxyuria triphosphate (dUTP)/deoxy hymidine triphosphate (dTTP) [9,10]. In addition, E165R may play an essential regulatory role in ASFV pathogenesis since its deficiency has been shown to significantly impair virus replication efficiency [11].

E165R is classified into the class I dUTPase family, which includes those from Homo sapiens [12], *Escherichia coli* (*E. coli*) [13], feline immunodeficiency virus (FIV) [14], equine infectious anemia virus (EIAV) [15], Mycobacterium tuberculosis [16], and white spot syndrome virus (WSSV) [17]. E165R is a homotrimeric dUTPase and contains five highly conserved motifs that exhibit active enzymatic sites with specific pyrophosphatase activity [11]. Interestingly, E165R contains a unique double-subunit active site and has extremely high tolerance to high temperatures [18]. Previous studies have shown that deletion of *E165R* significantly inhibits ASFV replication in vitro [11]. Therefore, E165R may serve as a potential drug target for inhibiting ASFV infection [10]. The availability of the high-resolution crystal structure of E165R has provided a basis for developing ASFV-related immunogenic drugs. However, the identification of epitopes that inhibit this enzyme is required.

In this study, we produced and examined a panel of 19 mAbs that specifically target E165R. Subsequently, we performed epitope mappings by expressing shortened overlapping polypeptides and synthesized oligopeptides. The epitopes were mainly located at the motif II, III, IV, and V of E165R (100–160 aa). Importantly, we identified a novel specific inhibitory antibody that can recognize an epitope in the motif V region. The serological characteristics of this antigenic region were evaluated and the potential therapeutic applications of these mAbs and epitopes were discussed.

## 2. Materials and Methods

### 2.1. Recombinant Plasmid Constructs for Protein Expression and Purification

The *E165R* gene (NCBI reference number: MK333180.1) was synthesized (Sangon Biotech Co, Shanghai, China) based on the genomic sequence of ASFV HLJ strain (Pig/HLJ/2018, GenBank: MK333180.1). Full-length or truncated sequences of E165R were amplified with specific primers (Table S1) using the synthesized gene as the template. PCR products were digested with *Nde* I and *Hin*d III restriction enzymes and then were cloned into pET-28a vector (Novagen, Darmstadt, Germany), expressing a hexahistidine (His) tag in its N terminal. Transformed *E. coli* colonies that carry the desired plasmid constructs were picked and grown in LB medium containing 30 μg/mL kanamycin to an optical density at 600 nm (OD_600_) of 0.5 to 0.6 at 37 °C. Protein expression was induced by 0.5 mM IPTG (Isopropyl-β-D-thiogalactopyranoside) at 16 °C, and the *E. coli* were harvested 16 h later. Harvested *E. coli* were lysed with lysis buffer (20 mM Tris-HCl, 150 mM NaCl, pH 8.5), and homogenized at low temperature using an ultrahigh-pressure *E. coli* disrupter (Antox Nanotechnology, Suzhou, China). The *E. coli* lysate was centrifuged at 20,000× *g* for 60 min at 4 °C to remove *E. coli* debris before being loaded in two batches onto a HisTrap FF (GE Healthcare, CA, USA) column equilibrated with lysis buffer. The column was washed three to five times with 10 mL of wash buffer containing 20 mM Tris-HCl (pH 8.5), 150 mM NaCl. Protein elution was achieved with elution buffer containing 20 mM Tris-HCl (pH 8.5), 150 mM NaCl, and 300 mM imidazole. Eluted protein was further purified using a HiLoad 16/600 Superdex 200 pg (GE Healthcare, CA, USA) column equilibrated with 20 mM Tris-HCl (pH 8.5) and 50 mM NaCl. Recombinant E165R protein was identified by Western blotting using anti-His-Tag MAB-HRP-Direct and the serums of pigs recovered from ASFV infection (CVCC NO: Z287; China Institute of Veterinary Drug Control, Beijing, China).

### 2.2. Enzyme Kinetics Measurement

According to previous reports, hydrolysis of dUTP by dUTPase is accompanied by the production of pyrophosphate (PP_i_) and H^+^ [9,18]. Cresol red is an agent commonly used in testing dUTPase enzyme activity because it can be protonated and deprotonated as an indicator with different absorption at 573 nm [19]. The reaction system comprises 10 nM protein, 50 μM dUTP, and 40 μM cresol red in a buffer system containing 2 mM bicine, 100 mM KCl, and 5 mM MgCl_2_ at pH 8.0, and incubated at a constant temperature of 25 °C for 2 min. UV absorbance was measured at 573 nm in a spectrophotometer using cuvettes with a 10 mm path length at a constant temperature of 25 °C and the change was analyzed using GraphPad Prism 9.3.0 (GraphPad Software, CA, USA). All measurements were carried out in at least three replicates.

### 2.3. mAbs Production and Purification

Purified protein was emulsified entirely with Freund’s immune adjuvant. BALB/c mice were immunized once every 2 weeks with 0.1 mg of protein 5 times. Antibody titer was detected in serum samples by ELISA with coated protein (1 μg/mL, 100 μL/well) after the fourth and fifth immunization. Splenocytes were isolated from mice with high antibody titer in the serum for fusion with SP2/0 murine myeloma cells. After two rounds of subclonal screening of fusion cells, 19 strains of mAbs were obtained. Female BALB/C mice aged 10 weeks were intraperitoneally injected with positive hybridoma cells (about 3 × 10^6^). After 7 days, the mice developed abdominal distension. Ascites were collected and centrifuged, and the supersuperant was ascites containing mAbs. The collected ascites were purified by ProA NUPharose FF (NO: NRPB02S; Hangzhou NeuroPeptide Biological science and Technology, Hangzhou, China).

### 2.4. Indirect ELISAs 

First, 96-well ELISA plates were coated with 100 μL of recombinant protein (1 µg/mL) or synthetic peptides (2 mg/mL) in carbonate coating buffer (pH 9.6) and incubated at room temperature (RT) for 3 h. Coated plates were washed once with 300 µL of PBST (0.05% Tween 20 in PBS) per well using a Biotek plate washer (BioTek Instruments Inc., VT, USA). Each well was blocked with 250 μL of 5% non-fat milk in PBS for 30 min at RT before being washed once with 300 μL of PBST and then incubated with 100 μL of testing sera (1:100 dilution in PBS) for 30 min at 37 °C. The serums of mice before immunization were used as the negative control in subclonal screening of fusion cells. In the identification of epitopes by polypeptide ELISA, we used *E. coli*-derived ASFV E120R protein as the negative control, and PBS as the no template control. Subsequently, each well was washed 3 times with 300 μL of PBST before incubation with 100 μL of HRP-conjugated goat anti-mouse secondary antibodies (NO: ab6789; Abcam, Cambridge, UK) (1:10,000 dilution) for 30 min at 37 °C. After washing 5 times with 300 μL of PBST, each well was added with 100 μL of TMB single-component substrate solution (NO: PR1200; Solarbio, Beijing, China) and then left at RT for 15 min of incubation. An equal volume of 1 M hydrochloric was added to terminate the reaction. Absorbance was measured at 450 nm in a SPARK 10 M plate reader (TECAN Inc., Männedorf, Switzerland). The results were expressed as optical density (OD). The average of the negative control values plus 3 times standard deviation was used as the negative control threshold. When the average of the measured OD_450_ value of the well was greater than the negative control threshold, it was considered as positive.

### 2.5. Western Blotting Assay

Proteins were separated on 12.5% SDS-PAGE gels and transferred onto PVDF membranes (Bio-Rad Laboratories, Hercules, CA, USA), which were then blocked for an hour in 5% non-fat milk in PBS. Blocked membranes were subsequently incubated with mouse anti-His tag monoclonal antibody (NO: M30111; Abmart, Shanghai, China) (1:5000) or swine anti-serum (1:1000) for 1 h at RT, followed by 3 washes, 10 min each, with PBST before incubation with goat anti-mouse HRP (1:5000) or goat anti-swine HRP-labeled secondary antibody (NO: ab102135; Abcam, Cambridge, UK) (1:5000) for 1 h at RT. Membranes were then washed three times before incubation with SuperSignal West Pico Chemiluminescence membrane substrate (Thermo Fisher Scientific, Waltham, MA, USA) for 5–10 mins. Signals were detected by an Amersham Imager 680 (General Electric, Schenectady, NY, USA).

### 2.6. Immunofluorescence Assay (IFA)

The full-length *E165R* gene was amplified by PCR using the synthesized construct as the template (Table S1). The PCR product was cloned into pCAGGS-HA vector (Addgene, Teddington, UK) using *Eco*R I and *Xho* I restriction sites. PK15 cells (porcine kidney cell) were transfected with 500 ng of plasmid per well in a 24 well plate. The culture medium was replaced with DMEM supplemented with 10% FBS at 6 h post transfection. The cells were fixed with 3% paraformaldehyde for 30 min before being washed 3 times with PBS and then incubated with anti-E165R mAb (1:500) or anti-HA tag mAb (NO: ab18181; Abcam, Cambridge, UK) (1:1000). After 1 h of incubation at 37 °C, cells were washed 3 times with PBS and subsequently incubated with Alexa Fluor-488-conjugated goat anti-mouse secondary antibody (NO: ab150113; Abcam, Cambridge, UK) (1:500) at 37 °C for 1 h. Following the washing step 3 times with PBS, the cells were observed under the OLYMPUSIX IX73 plore Standard fluorescence microscope (OLYMPUSIX, Tokyo, Japan).

### 2.7. Epitope Mapping of E165R

Epitopes were identified with mAbs by Western blotting and indirect ELISA assay. Based on the detection results, overlapping peptides of 6–7 amino acids (aa) were designed and synthesized (Genscript, Nanjing, China) to detect the epitopes specifically. ELISA plates were coated with 100 μL of overlapping peptides (100 µg/mL) in carbonated coating buffer (pH 9.6).

### 2.8. Testing of Inhibitory Antibody

To each well, 970 μL of buffer solution (2 mM Bicine, 150 mM KCl, 5 mM MgCl_2_, 40 μM cresol red pH 8.0), 10 μL of dUTPase (10 μM) and different volumes of anti-E165R-mAb (1 mg/mL) were added followed by incubation for 3 min. Enzyme reactions were initiated by adding 10 μL of dUTP (5 mM). Enzymatic activity was continuously detected for the production of PPi for 1 min with varying concentrations of anti-E165R-mAb and the UV absorbance at 573 nm was measured every 2 s. Data were analyzed by GraphPad Prism 9.3.0 to determine the inhibitory antibody, optimal dilution, and kinetic parameters of anti-E165R-mAb.

### 2.9. Monoclonal Antibody Isotyping

Mouse monoclonal antibody isotyping reagents (Catalog number: SEK003; Sino Biological, Beijing, China) were used to classify antibodies detected in the culture supernatant of monoclonal cells according to the manufacturer’s recommended instructions. Briefly, 100 μL of culture supernatant were added into each well, and after the signal was developed with TMB single-component substrate solution. The absorbance of each well was determined at 450 nm.

### 2.10. Sequence Alignment of Classical dUTPases

To analyze the conservation of identified epitopes, the corresponding amino acid sequences of epitopes from some ASFV strains from different countries or regions and other viruses were aligned by DNAstar MegAlign software 7.0 (DNASTAR, Inc., Madison, WI, USA).

### 2.11. Structure Analysis and Specificity Verification of Inhibitory Antibody-Recognized Epitope

PyMol software was used to simulate the catalytic structures of ASFV dUPase (PDB code: 6LJ3) and Sus scrofa dUPase (PDB code: 6LJJ). Details of the antibody recognition sites were displayed and compared in order to identify structural differences.

### 2.12. Data Analysis

Amino acid sequence alignment was performed by DNAstar MegAlign software 7.0 (DNASTAR lnc., WI, USA). The phylogenetic tree was drawn using MEGA-X software (version 10.2.6, Mega Limited, Auckland, New Zealand). All statistical differences were analyzed by one-way ANOVA using GraphPad Prism 9.3.0 software (GraphPad Software Inc., San Diego, CA, USA). A *p* value of <0.05 was considered to be statistically significant.

## 3. Results

### 3.1. Expression and Purification of the Recombinant E165R Protein

**Figure 1:**
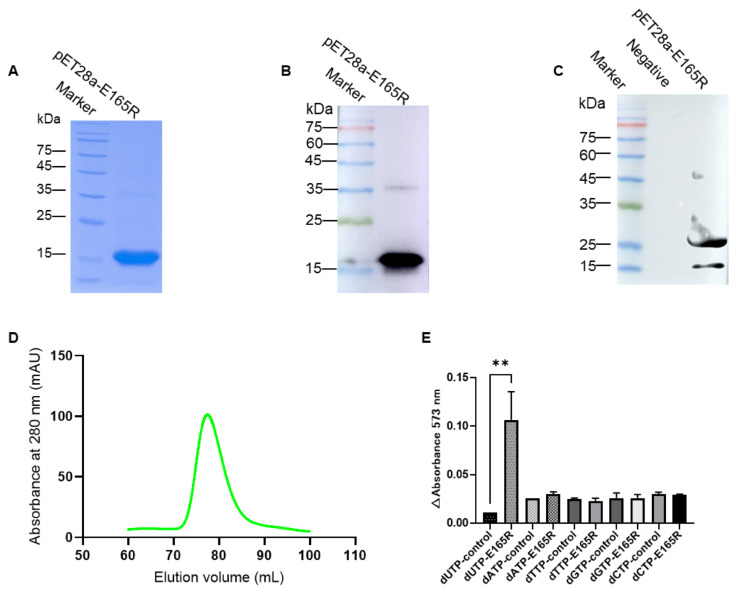
Identification of the immunogenicity and specificity of recombinant E165R (dUTPase) protein. (**A**) Analysis of purified E165R protein by HisTrap FF. (**B**) Identification of expressed recombinant E165R using mAb against His-tag by Western blotting. (**C**) Identification of expressed recombinant E165R using ASFV-positive serum by Western blotting. (**D**) Analysis of purified E165R protein by gel-filtration chromatography. (**E**) Enzyme activity and substrate specificity of full-length recombinant E165R (dUTPase). Note: “**” means the difference is significant at the 0.01 level.

SDS-PAGE analysis showed that E165R protein was expressed and purified successfully by affinity chromatography and gel-filtration chromatography with an expected size of 18.5 kDa (Figure 1A). Western blotting analysis using anti-His mAb confirmed that the His-tagged E165R recombinant protein was successfully expressed and purified (Figure 1B). Further analysis by Western blotting showed that the His-tagged E165R recombinant protein reacted with positive serum against ASFV, indicating that the protein has good antigenicity and the test results of the band greater than 25 kDa may be a non-specific reaction between ASFV-positive serum and *E. coli* self-expressed protein (Figure 1C).

Gel-filtration chromatography purification showed an obvious single peak in the 70–90 mL elution volume (Figure 1D). Functional identification by the pyrophosphate test confirmed that the E165R recombinant protein specifically degraded dUTP but not other ribonucleic acids (Figure 1E). These results indicate that the E165R recombinant protein has good enzymatic reactivity.

### 3.2. Production and Characterization of Anti-E165R mAbs

**Figure 2:**
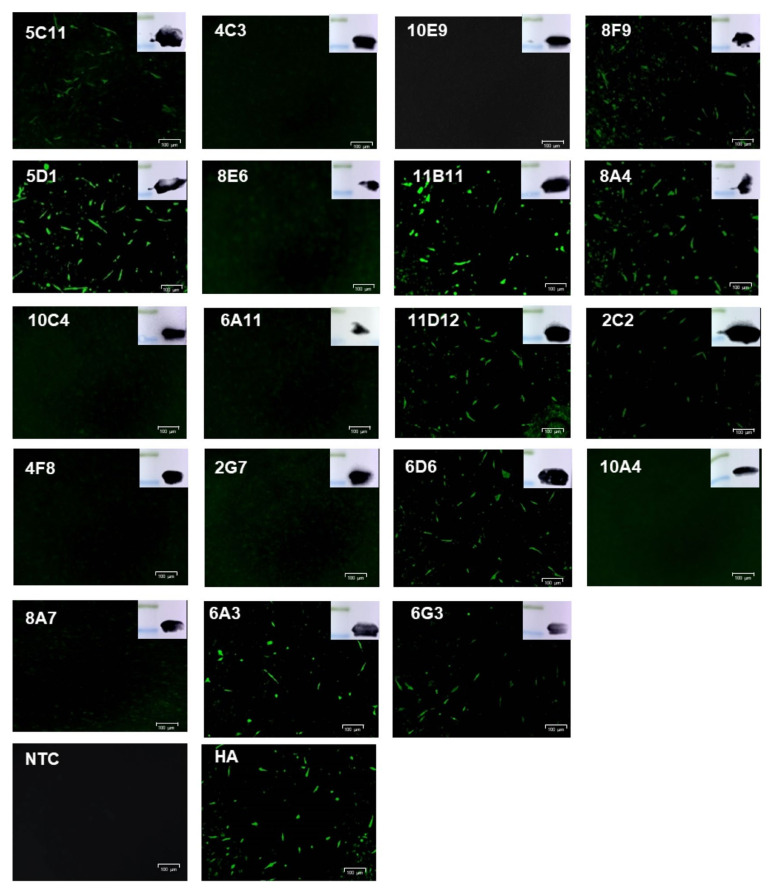
Western blotting analysis of recombinant His-E165R by Anti-E165R-mAbs and immunofluorescence detection of HA-E165R expression in PK15 cells by Anti-E165R-mAbs.

To prepare monoclonal antibodies against E165R, mice were immunized with purified E165R recombinant protein. Using traditional hybridoma cell fusion technology, a total of 19 positive monoclonal fusion cell lines were generated from two rounds of ELISA screening as confirmed by Western blotting (Table 1, Figure 2). To determine whether the mAbs can bind E165R protein expressed by the eukaryotic system, PK15 cells were transfected with pCAGGS-HA-E165R. IFA results showed that 10 out of 19 monoclonal antibodies could detect the recombinant E165R fusion protein at 72 h post-transfection (Figure 2). 

To investigate whether these mAbs could inhibit the enzyme activity of E165R, the E165R recombinant protein was incubated individually with each mAb, and the respective maximum reaction rates were calculated using the integrated Michaelis–Menten method [20]. Our results showed that 8F9, 5D1, 6A3, and 6G3 mAbs inhibited the enzymatic activity of E165R (Table 2), in which 8F9 showed the highest inhibition rate at 62.50%. 

To identify the mAb subtypes of 8F9, 5D1, 6A3, and 6G3, an antibody subtype classification kit was used. Our results showed that 8F9 and 5D1 mAbs are IgG2a, whereas 6A3 and 6G3 are IgG1 (Table 3).

### 3.3. Identification of Epitopes Recognized by the mAbs

**Figure 3:**
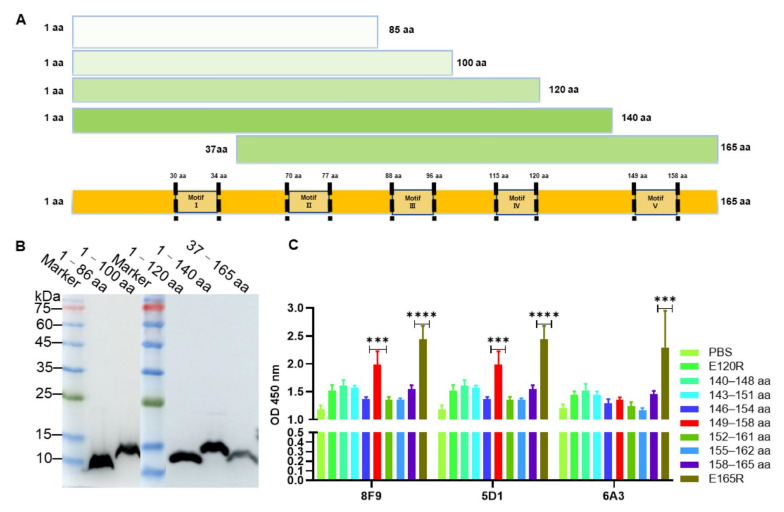
Expression of truncated recombinant proteins of ASFV dUTPase (E165R) and epitope identification of monoclonal antibody. (**A**) Schematic diagram showing the length and amino acid positions of truncated recombinant E165R proteins. (**B**) Identification of truncated recombinant E165R proteins using mAb against His-tag by Western blotting. (**C**) Identification of monoclonal antibody recognition epitopes by ELISA. Note: “***” means the difference is significant at the 0.001 level and “****” means the difference is significant at the 0.0001 level.

To identify the epitopes recognized by these mAbs, E165R was truncated into five fragments with amino acid residues spanning across positions 1–86, 1–100, 1–120, 1–140, and 37–165, respectively (Figure 3A). These truncated E165R proteins were also successfully expressed and purified with expected sizes using *E. coli* BL21 (Figure 3B). Western blotting analysis showed that the 140–165 aa, 120–140 aa, 100–120 aa, and 37–86 aa fragments were respectively recognized by 8, 6, 4, and 1 mAbs (Table 1, Figure S1). These results indicated that the antigenic sites of E165R are mainly distributed in the 100–165 aa region. Among these mAbs, three mAbs 8F9, 5G1, and 6A3 inhibited the enzyme activity and bound to the 140–165 aa region (Table 1). To further determine the epitopes recognized by these three mAbs, overlapping peptides with 6–7 aa were synthesized based on the amino acids spanning across positions 140–165 (Table S2). Using these peptides as coating antigens, the ELISA results showed that 8F9 and 5G1 specifically recognized the peptide sequence 149–RGEGRFGSTG–158 located in the motif V of E165R (Table 3).

### 3.4. Conservation of Epitopes Recognized by mAbs

**Figure 4:**
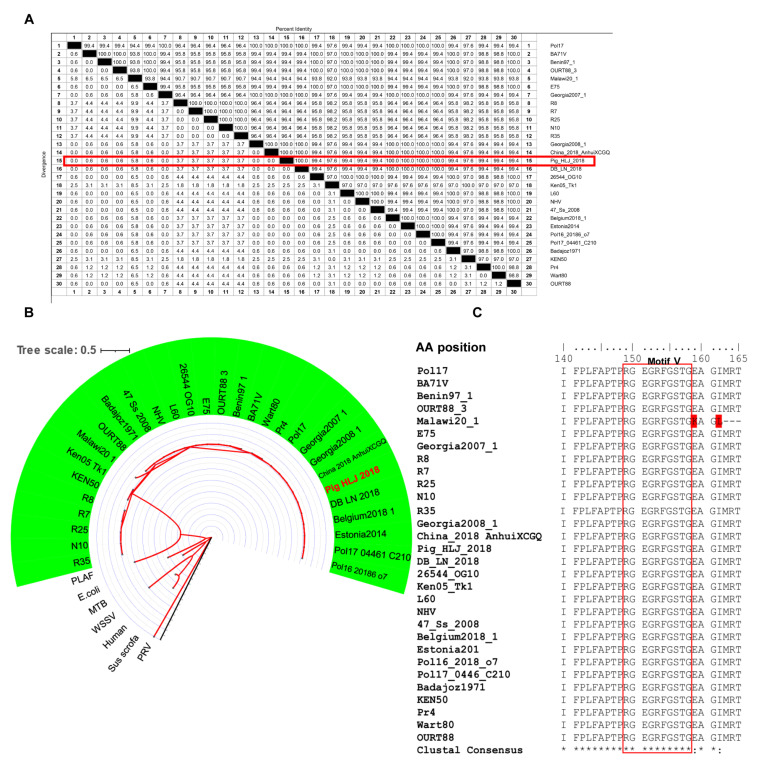
Homology analysis of the ASFV dUTPase (E165R) amino acid sequence. (**A**) Alignment of E165R amino acid sequences among different ASFV strains. The similarity between the amino acid sequence of E165R of Pig_HLJ_2018 and other ASFV strains is highlighted in red. (**B**) E165R genetic evolution analysis (maximum likelihood method (ML)). Human, Homo sapiens; PRV, Pseudorabies virus; PLAF, Plasmodium falciparum; WSSV, White spot syndrome virus; *E. coli*, *Escherichia coli*; MTB, Mycobacterium tuberculosis. The ASFV strain is shaded in green and sequence ID details are shown in Table S3. Branch lengths are highlighted in red. (**C**) Homology analysis of epitopes in E165R recognized by 8F9 mAb, residues in motif V are highlighted in red and differential amino acids are marked in red background.

The E165R amino acid sequences of the Pig/HLJ/2018 strain and other strains were found to share more than 94% homology (Figure 4A). Phylogenetic analysis showed that the dUTPase amino acids among different ASFV strains were highly conserved (Figure 4B). In particular, the motif V region of E165R was highly conserved among different ASFV strains (Figure 4C).

### 3.5. The Epitope on ASFV dUTPase Was Specifically Recognized by 8F9 mAb

**Figure 5:**
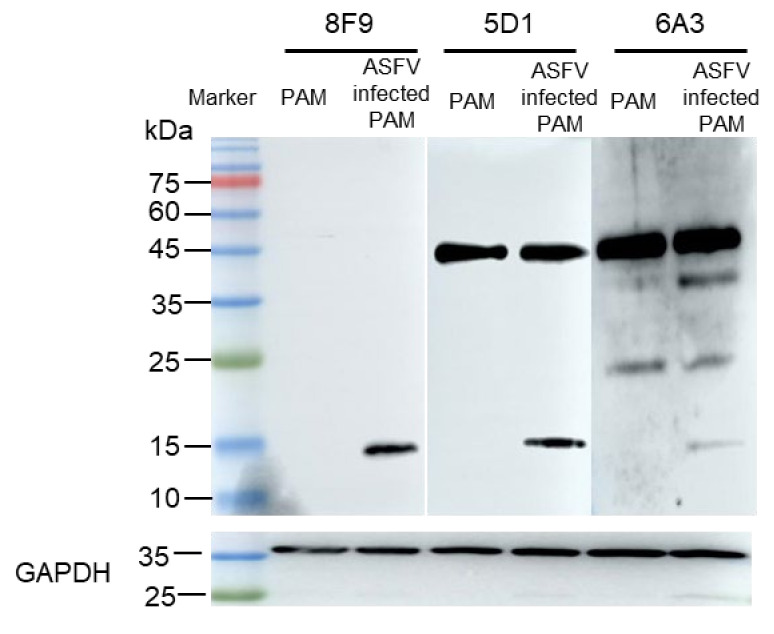
Antibody specificity verified by Western blotting using 8F6, 5G1, and 6A3 mAbs in PAMs and PAMs infected with ASFV.

To obtain monoclonal antibodies that can specifically recognize ASFV dUTPase, we used 8F9, 5G1, and 6A3 mAbs to detect porcine alveolar macrophages (PAMs) and ASFV-infected PAMs by Western blotting. Our results showed that 8F9, 5G1, and 6A3 specifically recognized ASFV dUTPase, and 5G1 and 6A3 had non-specific binding with protein of PAMs. Further, 6A3 may cross-react with Sus scrofa dUTPase (24.4 KDa) (Figure 5), indicating that the motif V epitope region of ASFV dUTPase is specific to 8F9 and 5G1 mAbs. Moreover, 8F9 specifically recognized ASFV dUTPase but not Sus scrofa dUTPase and protein of PAMs.

**Figure 6:**
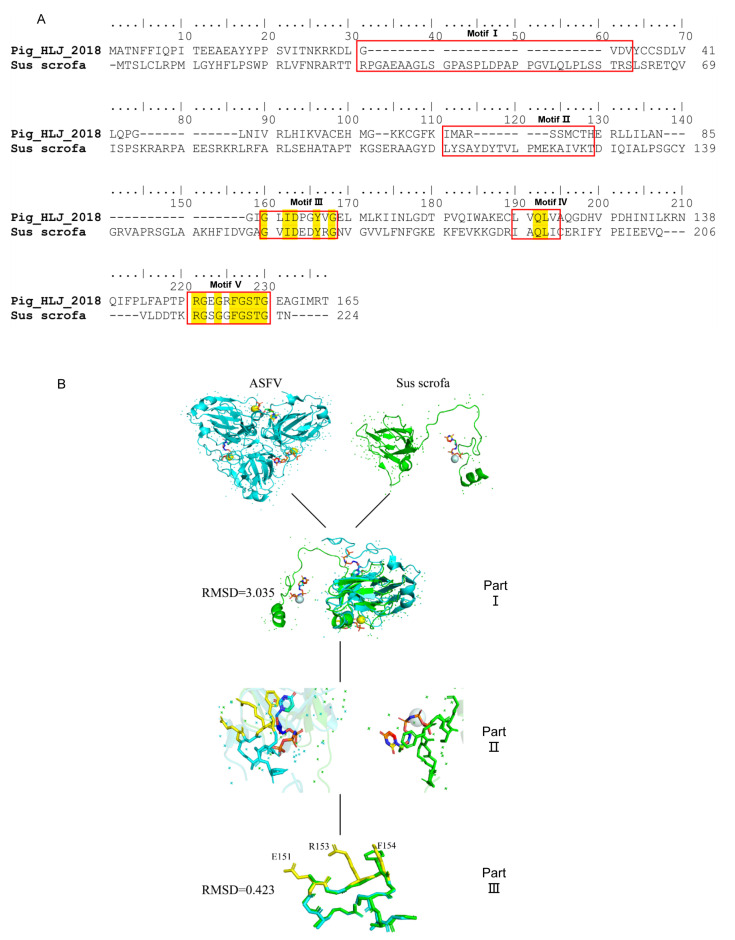
Specificity of 8F9 mAb in epitope recognition. (**A**) Amino acid sequence alignment of dUTPase from ASFV and Sus scrofa, where motif regions of dUTPase are highlighted in red, identical amino acid residues are highlighted in yellow. (**B**) Structure comparison of ASFV E165R with Sus scrofa dUTPase in complex with substrates. Part II: protomer of ASFV E165R is compared with that of Sus scrofa dUTPase. Part II: relative position amplification of neutralizing epitope of 8F6 mAb in part II. Part III: position of 8F6 mAb neutralizing epitope for ASFV E165R compared with that of Sus scrofa; amino acids with structural differences are highlighted in yellow. The ASFV E165R and Sus scrofa dUTPase protomers are colored in sky blue and green, respectively. ‘+’ denotes electron.

To further investigate the specificity of 8F9 mAb, structural similarities between ASFV and Sus scrofa dUTPases were analyzed. Our results showed that ASFV and Sus scrofa exhibit amino acid similarities in the motif III, IV, and V regions (Figure 6A). The motif V of ASFV dUTPase is a phosphate-bound loop region located at the C-terminal of the peptide, which is difficult to display in the reported structural models of ASFV dUTPase due to its poor electron density. We compared the motif V structure of ASFV dUTPase and Sus scrofa dUTPase. Based on the root mean square deviation (RMSD) values, the motif V regions of ASFV dUTPase (PDB code: 6LJ3) display a high level of similarity to that of Sus scrofa dUTPase (PDB code: 6LJJ) (RMSD = 0.423), while their amino acid positions F151, R153, and F154 exhibit significant differences (Figure 6B).

## 4. Discussion

Currently, there are no effective drugs and vaccines for ASF prevention and control [3,21]. Previous studies have proven that E165R acts as a dUTPase that efficiently contributes to ASFV genome replication in target host cells [11]. E165R is also an important target protein for ASFV-related therapeutics development. As the key enzyme for replicating many viruses [22], dUTPase plays a variety of roles during virus infection in host cells. For instance, PRV dUTPase (UL50) is required for virus replication [23] by inhibiting type I IFN signal transduction and by promoting virus immune evasion [22]. The same inhibitory effect on IFN signal transduction has also been observed in Epstein-Barr virus (EBV)-induced neuroinflammation [24,25]. Additionally, dUTPase encoded by herpes simplex virus 1 (HSV-1) and laryngotracheitis virus (ILTV, gallid alphaherpesvirus 1) has been shown to be an important virulence factor [26,27].

Previous studies have shown that dUTPase can be targeted by several molecules that can inhibit dUTPase activity efficiency by competing with substrates at enzyme catalytic sites [28,29]. In the course of cancer treatment, dUTPase normally prevents incorporation of dUTP and 5FU-nucleotide FdUTP into DNA and reduces the effectiveness of chemotherapy. The combined use of the new-generation dUTPase inhibitor of TAS-114 and 5Fu-nucleotide FdUTP can enhance the efficacy of chemotherapy in non-small-cell lung cancer and gastric cancer [30]. A similar effect was also observed in the study of 1, 2, 3-triazole-containing [31] and compound 26 [32] uracil derivatives, which dramatically enhanced the growth inhibition activity of 5-fluoro-2’-deoxyuridine against HeLa S3 cells in vitro. Oxaliplatin exerts a synergistic effect against advanced colorectal cancer in combination with 5-fluorouracil (5-FU) and with leucovorin in the p53-proficient colorectal cancer cell line HCT116 [33]. In the development of new antimalarial drugs, Trityl and diphenyl deoxyuridine derivatives [34,35] and their acyclic analogues [36] are potent and selective inhibitors for the Plasmodium falciparum dUTPase (PfdUTPase). The compounds LabMol-144 and LabMol-146 have demonstrated fair activity against multidrug-resistant (W2) and sensitive (3D7) parasites and presented good selectivity versus mammalian cells [37]. In addition, a Staphylococcus pathogenicity island repressor protein called StlSaPIbov1 (Stl) exhibits potent dUTPase inhibition in Mycobacteria [38]. In the study of compounds that inhibit viruses dUTPase, (1-(4-(tritylamino) butyl) pyrimidine-2,4 (1H,3H)-dionel) [39], an inhibitor compound of P. falciparum dUTPase has been shown to exhibit an inhibitory effect on the activity of ASFV dUTPase [18]. In addition, the antibody that binds and inhibits dUTPase has been used against EBV infection [40,41]. In this study, 8F9, 5D1, 6A3, and 6G3 mAbs against ASFV dUTPase were shown to inhibit the activity of E165R (Table 2). Furthermore, the 8F9 and 5D1 antibody’s epitope was identified to be located in the amino acid residues at positions 149–158 of E165R. Previous structural studies have shown that amino acid residues N85, G88, L89, I90, D91, Y94, M99, R71, S72, and Q120 are crucial for the enzymatic activity of E165R, among which S72, D91, and Q120 are relatively conserved in different viral species [9,10]. Our homology analysis confirmed that these amino acids were relatively conserved in different ASFV genotypes (Figure 4C). We also found that dUTPase activity is inhibited by mAbs through motif V targeting, suggesting that motif V may play an important role in dUTPase catalysis.

In this study, antigenic epitope mapping indicated that E165R has at least two epitope sites: 100–120 aa and 140–165 aa. Since E165R is highly expressed at the early stage of ASFV infection, it is essential to screen for high-affinity antibodies to establish a serological diagnostic method for early virus detection. However, it is important to verify the inhibitory effect of mAbs on E165R at the cellular and host levels during ASFV infection.

The motif V region of dUTPase is not only a dephosphorate group of dUTP, but also an important viral factor that determines the location of PRV and EBV [22,42]. Motif V is located at the C terminal of the polypeptide chain and it contains a P-loop-like (a phosphate-binding loop-like) motif [43]. During enzyme catalysis, motif V bypasses the second subunit and reaches the active center of the enzyme catalytic site formed by the second and the third subunit. In the hydrolytic dump, the C terminus (motif V) returns to an open state, conductive to PPi release. The dUTP hydrolyze process is changed from a disordered state to an ordered catalytic state by the motif V region [9,10,18]. Here, we found that the inhibitory epitopes recognized by mAbs of 8F9 and 5G1 are located in the motif V of E165R. Additionally, we showed that 8F9 only decreases the catalytic efficiency of dUTPase, and it does not inhibit the enzyme activity completely. These results suggest that antibody binding to motif V inhibits PPi release, which then disrupts the dUTPase catalytic process.

In conclusion, we screened and obtained the antigenic epitope map of E165R by using a panel of monoclonal antibodies. Interestingly, we identified an mAb that can directly inhibit the catalytic activity of E165R. This study provides a basis that may aid further development of ASFV dUTPase-associated mAb drugs.

## Figures and Tables

**Table 1:** Reactivity of anti-E165R mAbs generated in this study.

Anti-E165R mAb	Western Blotting						IFA	Epitope Region aa
	1–165 aa	1–86 aa	1–100 aa	1–120 aa	1–140 aa	37–165 aa		
5C11	+	−	−	−	−	+	+	140–165
4C3	+	−	−	−	+	+	−	120–140
10E9	+	−	−	+	+	+	−	100–120
8F9	+	−	−	−	−	+	+	140–165
5D1	+	−	−	−	−	+	+	140–165
8E6	+	−	−	+	+	+	−	100–120
11B11	+	−	−	−	−	+	+	140–165
8A4	+	−	−	−	−	+	+	140–165
10C4	+	−	−	−	−	+	−	140–165
6A11	+	+	+	+	+	+	−	37–86
11D12	+	−	−	−	−	+	+	140–165
2C2	+	−	−	+	+	+	+	100–120
4F8	+	−	−	−	+	+	−	120–140
2G7	+	−	−	−	+	+	−	120–140
6D6	+	−	−	−	+	+	+	120–140
10A4	+	−	−	−	+	+	−	100–120
8A7	+	−	−	−	+	+	−	120–140
6A3	+	−	−	−	−	+	+	140–165
6G3	+	−	−	−	+	+	+	120–140
NC	−	−	−	−	−	−	−	N/A

Note: ”+”= positive, “−”= negative, and “N/A”= Not applicable.

**Table 2:** Effects of mAbs on the enzyme activity of recombinant E165R.

Antibody Volume	Buffer	Mouse Negative Serum + E165R	8F9 + E165R	5D1 + E165R	6A3 + E165R	6G3 + E165R
0 μL	10^−52^	−1.6 × 10^−3^	−1.6 × 10^−3^	−1.6 × 10^−3^	−1.6 × 10^−3^	−1.6 × 10^−3^
20 μL	N/A	−1.6 × 10^−3^	−1.2 × 10^−3^	−1.3 × 10^−3^	−1.3 × 10^−3^	−1.2 × 10^−3^
40 μL	N/A	−1.6 × 10^−3^	−1.1 × 10^−3^	−1.1 × 10^−3^	−1.1 × 10^−3^	−1.2 × 10^−3^
100 μL	N/A	−1.6 × 10^−3^	−6.0 × 10^−4^	−8.0 × 10^−4^	−9.0 × 10^−4^	−8.0 × 10^−4^
Max inhibition enzyme activity ratio (%)	N/A	N/A	62.50	50.00	43.75	50.00

Note: the numbers in the table represent the slope values of the linear regression equation for fitting.

**Table 3:** Anti-E165R-mAbs isotyping and inhibitory epitope analysis.

Anti-E165R-mAbs	Antibody Subtype	Inhibitory Epitope
8F9	IgG2a	149–RGEGRFGSTG–158
5D1	IgG2a	149–RGEGRFGSTG–158
6A3	IgG1	Undefined
6G3	IgG1	N/A

Note: ”N/A” = Not applicable.

## Data Availability

All available data are presented in this manuscript.

## Supplementary Materials

The following are available online at https://www.mdpi.com/article/10.3390/v13112175/s1, Figure S1: Identification of epitope regions recognized by monoclonal antibodies by Western blotting, Table S1: PCR Primers, Table S2: Synthesized peptides for epitope identification, Table S3: dUTPase Sequence ID information details in genetic evolution analysis.
