# Supplementary material for: Identification and Characterization of a Novel Epitope of ASFV-Encoded dUTPase by Monoclonal Antibodies
Source: Viruses. 2021 Oct 28;13(11):2175. doi: 10.3390/v13112175 (PMC8620545; doi:10.3390/v13112175)
Supplement: Supplementary file 1 [file viruses-13-02175-s001.zip › viruses-1410312-supplementary.pdf]

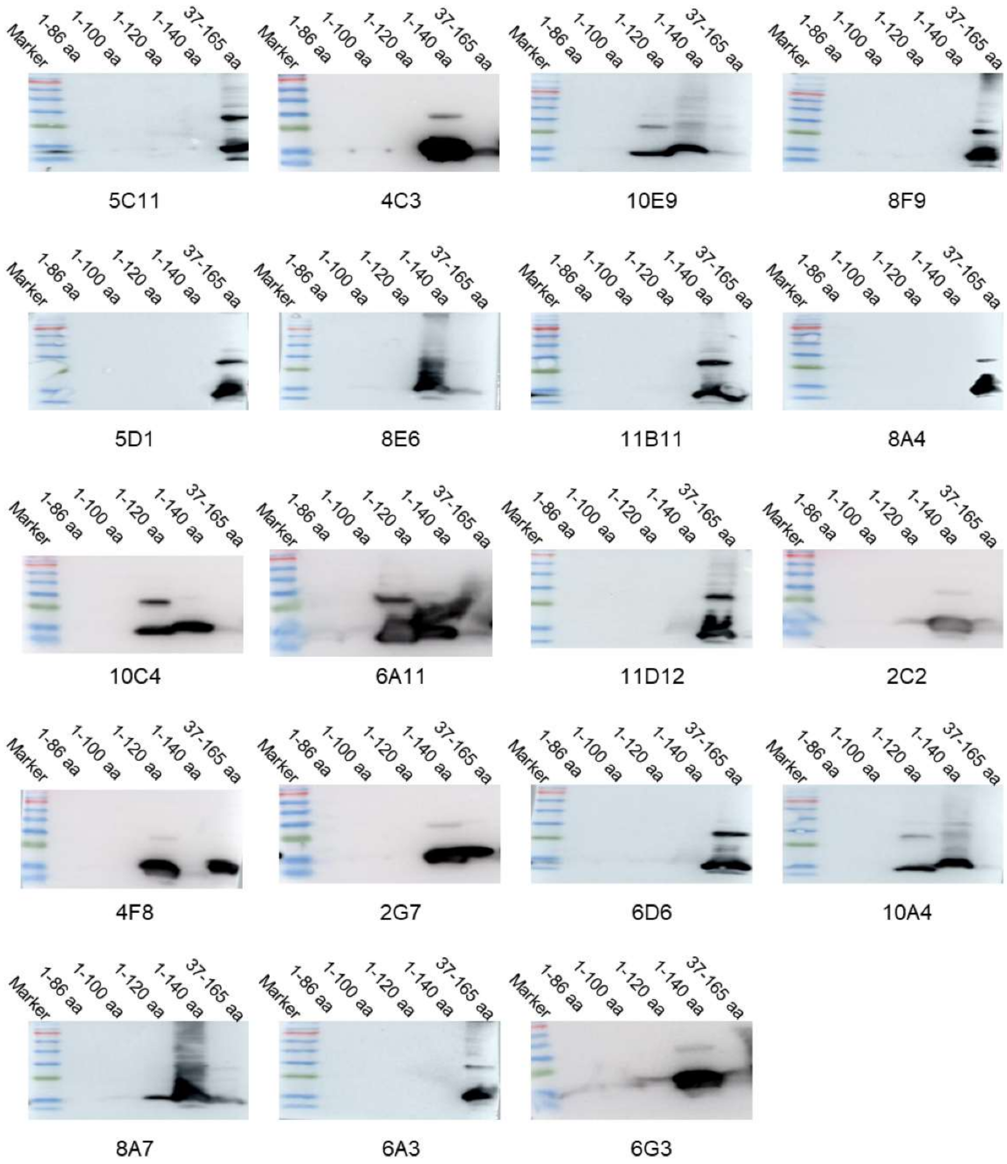

Figure S1 Identification of epitope regions recognized of monoclonal antibodies by western blotting.

Table S1 PCR Primers

Target primer	Sequence(5′-3′)	Bases(bp)
pET28a-E165R-*Nde* Ⅰ -1F	catatgATGGCAACAAATTTTTTTAT	26
pET28a- E165R-*Nde* Ⅰ -37F	catatgTGCTCCGACCTAGTGCTTCA	26
pET28a- E165R-*Hin*dIII-165R	aagcttTTAAGTTCTCATAATCCCGG	26
pET28a- E165R-*Hin*dIII-140R	aagcttTTATATTTGGTTTCTTTTTAGGA	26
pET28a- E165R-*Hin*dIII-120R	aagcttTTACTGCACCAAACATTCTTTGG	26
pET28a- E165R-*Hin*dIII-100R	aagcttTTAGAGCATGAGCTCGCCCACAT	26
pET28a- E165R-*Hin*dIII-86R	aagcttTTATCCGTTTGCAAGGATGAGCA	26
pCAGGS-HA-E165R-*Eco*R Ⅰ -F	gaattcATGGCAACAAATTTTTTTAT	26
pCAGGS-HA-E165R-*Xho* Ⅰ -R	ctcgagTTAAGTTCTCATAATCCCGG	26

Table S2 Synthesized peptides for epitope identification

Position(aa)	Peptide sequence
E165R-140-148	IFPLFAPTP
E165R-143-151	LFAPTPRGE
E165R-146-154	PTPRGEGRF
E165R-149-158	RGEGRFGSTG
E165R-152-160	GRFGSTGEA
E165R-155-163	GSTGEAGIM
E165R-158-165	GEAGIMRT

Table S3 dUTPase Sequence ID information details in genetic evolution analysis

Abbreviation	Full name	Sequence ID
Human	Homo sapiens	NP_001020419.1
PRV	Pseudorabies virus	AGW01097.1
PLAF	Plasmodium falciparum	XP_001347953.1
WSSV	White spot syndrome virus	YP_009220509.1
E.coil	Escherichia coli	WP_001298007.1
MTB	Mycobacterium tuberculosis	WP_003900552.1
Sus scrofa	Sus scrofa	A0A480PG39
Pol17	African swine fever virus isolate Pol17_04461_C210	AXZ96185.1"
BA71V	African swine fever virus strain BA71V	NP_042823.1
Benin97_1	African swine fever virus Benin 97/1 pathogenic isolate	CAN10230.1
OURT88_3	African swine fever virus OURT 88/3 (avirulent field isolate)	CAN10479.1
Malawi20_1	African swine fever virus Malawi LIL 20/1	Q65243.1
E75	African swine fever virus strain E75	CBH29230.1
Georgia 2007/1	African swine fever virus isolate ASFV Georgia 2007/1	CAD2068492.1
R8	African swine fever virus strain R8	AXB49359.1
R7	African swine fever virus strain R7	AXB49533.1
R25	African swine fever virus strain R25	AXB49705.1
N10	African swine fever virus strain N10	AXB49876.1
R35	African swine fever virus strain R35	AXB50049.1

Georgia 2008/1	African swine fever virus strain Georgia 2008/1	AZP54067.1
China/2018/AnhuiXCGQ	African swine fever virus isolate China/2018/AnhuiXCGQ	AYW34101.1
Pig/HLJ/2018	African swine fever virus isolate Pig/HLJ/2018	QBH90618.1
DB/LN/2018	African swine fever virus isolate DB/LN/2018	QBH90803.1
26544/OG10	African swine fever virus isolate 26544/OG10 from Italy	AJZ77148.1
Ken05/Tk1	African swine fever virus strain Ken05/Tk1	AJL34143.1
L60	African swine fever virus strain L60	AIY22321.1
47/Ss/2008	African swine fever virus isolate 47/Ss/2008	AOO54500.1
NHV	African swine fever virus strain NHV	AIY22479.1
Belgium 2018/1	African swine fever virus isolate ASFV Belgium 2018/1	VFV48064.1
Estonia 2014	African swine fever virus isolate Estonia 2014	SPS73553.1
Pol16_20186_o7	African swine fever virus isolate Pol16_20186_o7	AXZ95902.1
Pol17_04461_C210	African swine fever virus isolate Pol17_04461_C210	AXZ96185.1
Pol17_03029_C201	African swine fever virus isolate Pol17_03029_C201	MG939587.1
KEN 50	African swine fever virus isolate Kenya_1950	AY261360.1
Wart80	African swine fever virus isolate Namibia_Warthog_1980	AY261366.1
Pr4	African swine fever virus tick/South Africa/Pretoriuskop Pr4/1996	UniProtKB/Swiss-Prot: P0C9C3.1
OURT88	African swine fever virus (isolate Pig/Portugal/OURT88/1988) (ASFV)	UniProt: A9JM59
